# Different Preclimacteric Events in Apple Cultivars with Modified Ripening Physiology

**DOI:** 10.3389/fpls.2017.01502

**Published:** 2017-09-05

**Authors:** Vikram Singh, Asya Weksler, Haya Friedman

**Affiliations:** Department of Postharvest Science of Fresh Produce, Agricultural Research Organization, Volcani Center Bet Dagan, Israel

**Keywords:** “Anna”, developmental regulators, ethylene biosynthesis, fruit development, MdFUL, system I/II, MdCNR, respiration

## Abstract

“Anna” is an early season apple cultivar exhibiting a fast softening and juiciness loss during storage, in comparison to two mid-late season cultivars “Galaxy” and “GD.” The poor storage capacity of “Anna” was correlated with high lipid oxidation-related autoluminescence, high respiration and ethylene production rates, associated with high expression of *MdACO1, 2, 4, 7*, and *MdACS1*. All cultivars at harvest responded to exogenous ethylene by enhancing ethylene production, typical of system-II. The contribution of pre-climacteric events to the poor storage capacity of “Anna” was examined by comparing respiration and ethylene production rates, response to exogenous ethylene, expression of genes responsible for ethylene biosynthesis and response, and developmental regulators in the three cultivars throughout fruit development. In contrast to the “Galaxy” and “GD,” “Anna” showed higher ethylene production and respiration rates during fruit development, and exhibited auto-stimulatory (system II-like) effect in response to exogenous ethylene. The higher ethylene production rate in “Anna” was correlated with higher expression of ethylene biosynthesis genes, *MdACS3a MdACO2, 4*, and *7* during early fruit development. The expression of negative regulators of ripening (*AP2/ERF*) and ethylene response pathway, (*MdETR1,2* and *MdCTR1*) was lower in “Anna” in comparison to the other two cultivars throughout development and ripening. Similar pattern of gene expression was found for SQUAMOSA promoter binding protein (SBP)-box genes, including *MdCNR* and for *MdFUL*. Taken together, this study provides new understanding on pre-climacteric events in “Anna” that might affect its ripening behavior and physiology following storage.

## Introduction

Apple (*Malus* × *domestica*, subfamily; *Maloideae*, family; *Rosaceae*) is a classical climacteric fruit, generating an ethylene burst at the onset of ripening concomitantly with an increase in respiration (Alexander and Grierson, 2002; Giovannoni, 2004). One of the major concerns of apple fruit is their quality loss during storage, where ethylene is a major factor affecting the storage performance and is responsible for the changes in fruit texture and firmness loss. Apple cultivars vary considerably in their physico-chemical characteristics, texture, and storage performance (Hoehn et al., 2003; Johnston et al., 2009). The spring or summer apple cultivars like “Anna” (Pre-Aymard et al., 2003; Trainin et al., 2016), “Sunrise” (Wiersma et al., 2007), “McIntosh” (Harb et al., 2012), and “Gala” (Jenny et al., 1995) are prone to fast ripening and softening, however, mid-late season cultivars such as “Honeycrisp” (Harb et al., 2012), “Golden Delicious (GD)” (Wiersma et al., 2007), and “Fuji” (Wakasa et al., 2006; Wei et al., 2010) have long storage capacity and slow softening. “Anna,” an early maturating apple cultivar, which was developed in Israel, is becoming increasingly popular because of its low chilling requirement for flowering and a short fruit developmental period (Trainin et al., 2016). Nevertheless, “Anna” exhibits fast ripening, inability to maintain the crisp texture and becomes mealy, and hence has a poor storage capacity (even at 0°C) (Klein and Lurie, 1990). The reasons for this peculiar attributes of “Anna” are still not understood and the mechanism of ethylene biosynthesis and response is yet to be identified.

The ethylene biosynthesis is catalyzed by two major enzymes 1-aminocyclopropane-1-carboxylate synthase (ACS) and 1-aminocyclopropane-1-carboxylic acid oxidase (ACO). 1-Aminocyclopropane-1-carboxylate synthase catalyzes the rate-limiting step of the pathway leading to the production of the ethylene intermediate, 1-aminocyclopropane-1-carboxylic acid (ACC) from *S*-adenosyl-L-methionine (SAM), which then converted to ethylene by ACO (Alexander and Grierson, 2002). Each of these enzymes is encoded by a gene family, and is expressed differentaily during fruit ripening (Lin et al., 2009). Ethylene can modulate its own production by either positive or negative feedback regulation. Two main ethylene production systems (I and II) were characterized during tomato fruit development and maturity, where system I is auto-inhibitory and persists at early fruit development, however, system II is auto-stimulatory and is activated during ripening (Barry et al., 2000). Recently, based on expression data of various genes within *ACO* and *ACS* families in tomato, it was suggested that a third system exists at the decline of ethylene biosynthesis peak (Van de Poel et al., 2012).

In apple genome, a total of 19 *MdACS* genes have been identified (Li et al., 2013), and among these only *MdACS1, 3a, 5A, 5B*, and *6*–*9* were expressed specifically in fruit cortex, possibly playing a role in fruit ripening (Li et al., 2013). Both, *MdACS3a* and *MdACS6* are expressed at early fruit development stages, and it was suggested that *MdACS6* is responsible for increased *MdACS3a* expression (Li et al., 2015), and a null mutation in *MdACS3* correlated with increase shelf life (Wang et al., 2009). On the other hand, *MdACS1* is expressed concomitantly with fruit ripening (system II) and most likely is responsible for the burst of ethylene production, and indeed, transgenic apple fruit silenced in *MdACS1*, are blocked in ethylene production (Dandekar et al., 2004). In accordance with *MdACS1* participation in system II, its expression was blocked by 1-methylcyclopropene (1-MCP) and enhanced by ethephon (ethylene inducer) (Tan et al., 2013), and on the other hand, the expression of *MdACS3a* is upregulated or remain unchanged by 1-MCP, and is inhibited by ethephon treatment, fitting with its involvement in system I of ethylene production (Varanasi et al., 2011; Tan et al., 2013), similarly to *SlACS6* in tomato (Nakatsuka et al., 1998). In contrast to *ACS*, smaller differences in the expression of *ACO* genes were observed during fruit development in many plant species, including tomato (Nakatsuka et al., 1998; Barry et al., 2000) and apple (Dong et al., 1992). In apple, seven, *MdACO1*–*7* have been identified (Clouse and Carraro, 2014), but only *MdACO1*–*4* were found to be expressed in fruit. Specifically, *MdACO1* plays a role in auto-stimulatory ethylene production (system II), and silencing of this gene, inhibited ethylene production (Schaffer et al., 2007). Although various ethylene biosynthesis genes affecting apple ripening have been identified, the physiological and molecular mechanism(s) involved in the transition from system I to II are still unknown, and it is not clear if all cultivars possess similar mechanism(s).

Ethylene action is executed via signal transduction pathway involving membrane-localized receptors and kinase cascade, which act as negative regulators in ethylene response. In apple, the ethylene receptor (ETR) are encoded by nine different genes *MdETR1, 1b, MdETR2, MdETR5, MdERS1, MdERS2* (Cin et al., 2005; Tatsuki et al., 2007; Wiersma et al., 2007), *MdETR101, MdETR105*, and *MdETR102* (Ireland et al., 2012). The expression of these receptors is induced during ripening and few are induced by ethylene treatment (Tatsuki et al., 2009; Ireland et al., 2012; Yang et al., 2013). The constitutive triple response-1 (CTR1), *MdCTR1* acts downstream from the ETRs, and its gene expression is upregulated by ethylene during fruit ripening (Wiersma et al., 2007; Yang et al., 2013). The negative regulators, ETRs and *CTR1* were increased during tomato fruit ripening, and it was suggested that their higher expression modulates the sudden increase in ethylene concentration (Klee, 2002). The signaling process downstream of CTR1 involves the activation of positive regulator ethylene insensitive (EIN2, EIN3, and EIN3-like, EIL transcription factors), which activate mainly ethylene response factor/ethylene-responsive element binding protein (ERF/EREBP) (Cara and Giovannoni, 2008). These proteins act as *cis*-acting regulator for the ethylene responsive genes at the last step of ethylene signaling pathway. The increase in expression of these positive regulators *MdEIN2, MdEIL1, MdERF1*, and *2* during ripening or to external ethylene supply suggested their direct involvement in ethylene induction during ripening (Yang et al., 2013). In apple, *MdERF1, 2*, and *3* are involved in fruit ripening, and act specifically in *ACS* regulation (Wang et al., 2007; Li et al., 2016) and *MdERF2* specifically regulates the expression of *MdACS3a* (Li et al., 2015). All these components affect ethylene response, but their contribution to apple fruit quality has not been investigated.

Ripening is the last stage of fruit development and events occurring at early stages might be required for transition toward ripening (Klee and Giovannoni, 2011). A three-component model was proposed (Zhong et al., 2013) for the transition from tomato fruit development to ripening is regulated by (a) unknown interacting mechanisms of ethylene with transcription factors, (b) fruit-specific transcription factors, and (c) epigenome reprogramming. All these can be modulated in different apple cultivars to affect fruit ripening. It has been known for a while that ethylene application at early fruit development hasten fruit ripening (Yang, 1987), and recently it has been demonstrated that ERFs modulate the time to ripening (Liu et al., 2015). In tomato many ripening-associated positive/negative transcription regulators have been identified including: MADS-box transcription factor ripening inhibitor (RIN), non-ripening (NOR), fruitful (FUL), colorless non-ripening (CNR), HD-Zip homeobox protein (HB-1), non-ripening (NR), tomato agamous-like1 (TAGL1), APETALA2a (AP2a), and SQUAMOSA promoter binding protein (SPB) which affect the transition to ripening (Leseberg et al., 2008; Klee and Giovannoni, 2011; Liu et al., 2015). In apple, *SEPALLATA1/2*-like (*SEP*-like) gene, *RIN* (*MdMADS8/9*) (Ireland et al., 2013) is involved in fruit ripening and *MdFUL* (*MdMADS2.1*) is associated with fruit firmness (Cevik et al., 2010). However, it is still not clear if these components are related to difference in apple qualities of various cultivars.

Therefore, this study was aimed to understand the ripening behavior of “Anna,” by comparing events at pre-climacteric stage to those in “Galaxy” and “GD.” Respiration, ethylene production, response to exogenous ethylene, and expression of genes involved in ethylene biosynthesis and response, and developmental regulators were compared between the cultivars at maturity, as well as, during fruit development.

## Materials and Methods

Apple fruit “Anna,” “Galaxy,” and “GD” were grown in two commercial orchards, “Anna” in Arugot (31°43′58.07″N; 34°46′34.46″E; altitude-72.6 m), and “Galaxy” and “GD” in Havat-Matityahu (33°3′32.32″N; 35°25′59.65″E; altitude-745.5 m). All cultivars trees were kept at about 2 m height in a central leader architecture. Crop load for “Anna” was approximately 400–470 fruit/tree, while for “GD” and “Galaxy” 300–350 fruit/tree. Experiments were conducted during the years 2013–2015 with similar results and data presented here are of 2015. “Anna” blooms on mid-March with the commercial harvest on end of June, while “Galaxy” and “GD” blooms on mid-April and having their commercial harvest on mid-September. Fruit were collected at different developmental stages: stages 1–6 (S1–S6), where S6 is the commercial harvest (H) time. Samples for “Anna” were collected every 18 days after full bloom while those for Galaxy and GD every 25 days to fit the shorter developmental time of Anna. Fruit of S1–S6 were taken immediately for further analysis as detailed below. Fruit of S6/H were also stored for 1 (removal 1, R1) or 2 months (removal 2, R2). At each stage, from S1 to S5, S6/H, R1, and R2, index of absorbance difference (*I*_AD_), starch content, ethylene and CO_2_ production rates, and gene expression analysis were performed. Firmness (by penetration or deformation), luminescence, juice content, total soluble solids (TSSs), and titratable acidity (TA) were determined at S6/H, R1, and R2. For gene expression, fruit were sliced as a wedge from two opposite sides, peel and core were removed, tissue was frozen in liquid nitrogen and stored at -80°C, RNA was extracted from three biological replicated, each containing five fruit.

### Determination of Starch Content

Starch content was determined according to the protocol described (Blanpied and Silsby, 1992). Six apple fruit were sliced horizontally to make a disk covering the whole apple core, cortex, and peel. The exposed side of the apple was dipped into starch–iodine solution for 2 min. Flesh containing starch turn into blue–black color and were indexed from 1 to 8, where index 1 indicated high starch with dark blue–black color covering core and cortex tissue, and index 8 indicated no starch, with no color.

### Chlorophyll Measurement

Chlorophyll content was measured by portable delta absorbance (DA) meter (Sinteleia, Bologna, Italy). Delta absorbance meter measures the difference in absorbance of chlorophyll a, at 670 and 720 nm and expresses as index of absorbance difference (*I*_AD_ = A670–A720) (Costamagna et al., 2013). *I*_AD_ was measured for total 10 apples on two opposite sides of each fruit. Delta absorbance meter has been used previously to determine harvest time in apple (DeLong et al., 2014).

#### Ethylene Production and Respiration Rate

Fruit from S1 to S2 and S3 to S4 were placed in 120 mL and 600 mL jars, respectively, for 2 h at 20°C, and fruit of S5, S6/H, R1, and R2 stages were placed in 2 L jar for 1 h at 20°C. Following incubation, gas samples were collected and injected in gas chromatography for ethylene (C_2_H_4_) and respiration (CO_2_) measurement. Ethylene production was measured by (Varian 3300, United States) gas chromatography with alumina column, using FID detector, and CO_2_ concentration was measured by GC series 580 (GOW-MAC, United States) with a Poropak N column, using TCD-FID detector. The rate of respiration and ethylene was calculated as milliliter per kilogram per hour and microliter per kilogram per hour, respectively.

### Ethylene Treatment

The response to exogenous ethylene was performed at all developmental stages (S1–S5, S6/H). Ethylene (10 ppm) was supplied by injecting into airtight container, containing 10 fruit, for 24 h at 20°C. Following treatment, fruit were placed individually into 2 L jars for 1 h at 20°C, and the ethylene production and respiration rates were monitored as described above. In parallel, non-treated fruit were also examined similarly.

### Determination of Total Soluble Solids (TSS), Titratable Acidity (TA), Expressible Juice, and Firmness

For the determination of TSSs and TA content, each fruit (10 fruit per stage) was peeled and cut from opposite sides to make wedge-shaped slices. Slices obtained from each fruit were pooled and grounded, and the extracted juice was used for further analysis. Total soluble solid content was determined by digital refractometer PR-1 (Atago, Tokyo, Japan), and TA content was determined by the titration of 2 mL juice to 0.1 N NaOH (pH 8.2) using Dosimat 665 (Metrohm, Switzerland) with 678 EP/KF processors, and were expressed as percentage of malic acid.

Juice content (expressible juice) in the fruit was determined on a cylinder of 1 × 1 cm in length and diameter from the fruit cortex tissue, having the approximate weight of 2 g. The cylinder was weighed and compressed in 5 mL syringe, the extract was collected into Eppendorf tube, and centrifuged at 10,000 × *g* for 15 min. The amount of expressible juice was calculated as percent of original cylinder weight following the protocol of Lill and Van der Mespel (1988).

Fruit firmness was measured on 10 fruit, by penetration using a Agrosta^®^14 Motorized Digital Penetrometer or by deformation using Universal testing machine Inspekt table *blue* 5 kN (Hegewald & Peschke MPT GmbH, Germany). For penetrometer measurement, each fruit was peeled from two opposite side, and a probe (8 mm in diameter) was used to puncture the tissue for 40 ms. For deformation, force was applied to deform the apple by 5% of its circumference. The force applied for both, penetration or deformation was expressed in Newton (N).

### Autoluminescence Imaging

Autoluminescence emission is generated from spontaneous photons emission by oxidation of lipid molecules from the tissue (Birtic et al., 2011). This was determined by In-Vivo Imaging Systems (IVIS, PerkinElmer, MA, United States) on six fruit, sampled at S6/H, R1, and R2. Before acquiring the images, fruit were kept in dark for 24 h to avoid any photon excitation. All the imaging parameters were standardized for apple fruit, and were kept the same for all measurements. Autoluminescence from the samples was acquired using the following settings: f/stop = 1.2, binning = large, exposure time = 25 min, excitation = block, emission > 600 nm. Luminescence images showing emission in photons/s/cm^2^/steradian were captured and quantized from whole apples.

### RNA Extraction and Gene Expression Analysis

Total RNA was extracted using Spectrum^TM^ Plant Total RNA Kit (Sigma–Aldrich). The DNA contamination from RNA samples was removed by using TURBO DNA-*free*^TM^ kit (Ambion, Life Technology), subsequently, c-DNA was prepared using the Verso cDNA Synthesis Kit (Thermo Scientific), and was used for further analysis. Gene expression analysis was performed by quantitative reverse transcriptase-PCR (qRT-PCR) containing cDNA, forward and reverse primers, and Fast SYBR^TM^ (Applied Biosystems) in a 10 μL reaction volume. Reactions were performed into StepOnePlus Real-Time PCR System (Applied Biosystem) using reaction condition of 40 cycles for 10 s at 95°C, 15 s at 60°C, and 20 s for 72°C, and results were analyzed by StepOne Software. The relative expression levels of the targeted genes were calculated by either 2^-ΔΔC_t_^ or 2^-ΔC_t_^ method, using actin as housekeeping gene. Primers were designed using Primer3Plus and are listed in Supplementary Table 2.

### Fluidigm Analysis

High throughput gene expression analysis was performed using Biomark HD System (Fluidigm, United States). The Fluidigm 48.48 dynamic array chip was used following the manufacturer’s ADP37 Fast GE^1^ protocol, which allows 2304 simultaneous real-time PCR gene expression. Primer specificity and reference genes were validated prior to analysis. Pre-amplification of cDNA was performed on 1.25 μL of 50 ng μL^-1^ samples using Fluidigm PreAmp Master Mix (Fluidigm, PN 1005581), and 2.7 μL of each pre-amplified cDNA was mixed with 3 μL of SsoFast EvaGreen Supermix with Low Rox (BioRad, PN 1725211) and to 0.3 μL of 20× Binding Dye Sample Loading Reagent (Fluidigm, PN 1001388). Individual primer pairs (50 μM) in a 1.08 μL volume mixed with 3 μL Assay Loading Reagent (Fluidigm, PN 85000736) and 1.92 μL of Low TE. Total 5 μL of each sample mix or each assay mix was then pipetted into individual sample inlet in the 48.48 Dynamic Array chip, and an (IFC) controller MX (Fluidigm) to prime the chip. The loaded chip was placed in the BioMark system for PCR at 95°C for 10 min, followed by 40 cycles at 95°C for 15 s and 60°C for 1 min. Following each reaction in a specific inlet, the PCR amplification curve was generated and chip was imaged. The dynamic array raw data were analyzed with the Fluidigm Real-Time PCR Analysis software. The gene expression was calculated using the 2^-ΔC_T_^ method, following normalization with actin. Heatmap was prepared by MultiExperiment Viewer, MeV v4.9 software, using expression profile obtained from 2^-ΔC_t_^, where hierarchical clustering of genes was based on the Spearman correlation, allowing genes clustering according to their expression patterns.

### Statistical Analysis

Statistical analysis of data was performed by Tukey’s HSD pairwise comparison test at *p* ≤ 0.05, using JMP 5.0.1a statistical software (SAS Institute Inc., NC, United States).

## Results

### Fruit Quality Parameters at Harvest and Following Storage

**FIGURE 1:**
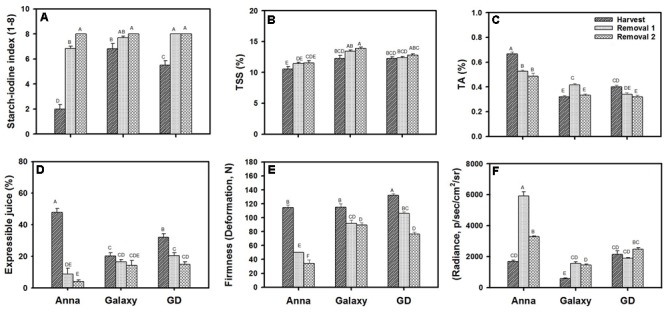
Quality parameters of “Anna,” “Galaxy,” and “GD” at harvest and following postharvest storage of 1 month (removal 1, R1) and 2 months (removal 2, R2). Fruit were stored at 0°C and analyzed following transferring to 20°C for 2 days. **(A)** Starch content, determined by starch–iodine index, **(B)** total soluble solids (TSS), **(C)** titratable acidity (TA), **(D)** expressible juice, and **(E)** firmness determined by 5% deformation, **(F)** lipid oxidation determined by autoluminescence and expressed in radiance (p/s/cm^2^/sr). Details of analysis are described in materials and methods. Parameters **(A–E)** represent the mean of 10 fruit, **(F)** with 6 fruit, and vertical bars indicate ±SE. Significance analysis was performed by comparing all cultivars and their stages collectively, using Tukey’s HSD pairwise comparison tests at *p* ≤ 0.05. Unlike letters represent significantly different groups.

We compared the quality parameters of “Anna” to “Galaxy” and “GD” at commercial harvest (S6/H) and following postharvest storage of 1 (R1) or 2 months (R2) (**Figure 1**). All cultivars achieved approximately similar size and weight at their commercial harvest (Supplementary Figure S1). Starch was highest at harvest of “Anna” in comparison to “Galaxy” and “GD.” For all three cultivars, the content of starch declined at R1–R2, showing no starch at R2 (**Figure 1A**).

A significant increase in TSS from H to R2 was observed only for “Galaxy” and the level was higher than in other cultivars (**Figure 1B**). “Anna” contained the highest TA content (**Figure 1C**), it decreased significantly from H to R2, with the major decline in “Anna,” compared to “GD” and “Galaxy.”

Expressible juice content was high at harvest for all cultivars, and declined significantly at R1 and R2 for “Anna” and “GD” (**Figure 1D**). “Anna” produced higher expressible juice at harvest than the other cultivars, showing a drastic decline at R1 (by 81%) and further at R2 (by 91%), whereas in “GD” decline was only by 36–53% at R1–R2, and “Galaxy” exhibited a marginal reduction of 18–30% at R1–R2. The juice levels following storage were high in “GD” and “Galaxy,” and therefore these cultivars remained juicier compared to “Anna” which developed mealiness after storage.

Fruit firmness which was measured, either by deformation (**Figure 1E**) or penetration (Supplementary Figure S2) was highest at H and declined at storage, R1 (56%) and R2 (70%) in “Anna” (**Figure 1E**). Comparatively, “Galaxy” and “GD” maintained fruit firmness with a reduction of 20–22% and 20–42% at R1–R2, respectively. Similar high reduction in firmness in “Anna” compared to the other cultivars was measured by penetration (Supplementary Figure S2).

The oxidative stress status of apple fruit was determined by measuring autoluminescence exerted from *in vivo* oxidation of lipids (**Figure 1F**). “Anna” and “GD” displayed high autoluminescence at harvest compared to “Galaxy.” Autoluminescence was enhanced significantly during storage for “Anna,” R1 (251%) and R2 (95%), but only by 168% and 148% for “Galaxy” at R1 and R2, respectively. Comparatively, “GD” showed a 12% reduction at R1 and 15% increment at R2.

### Ethylene Production and Respiration during Harvest and Storage

**FIGURE 2:**
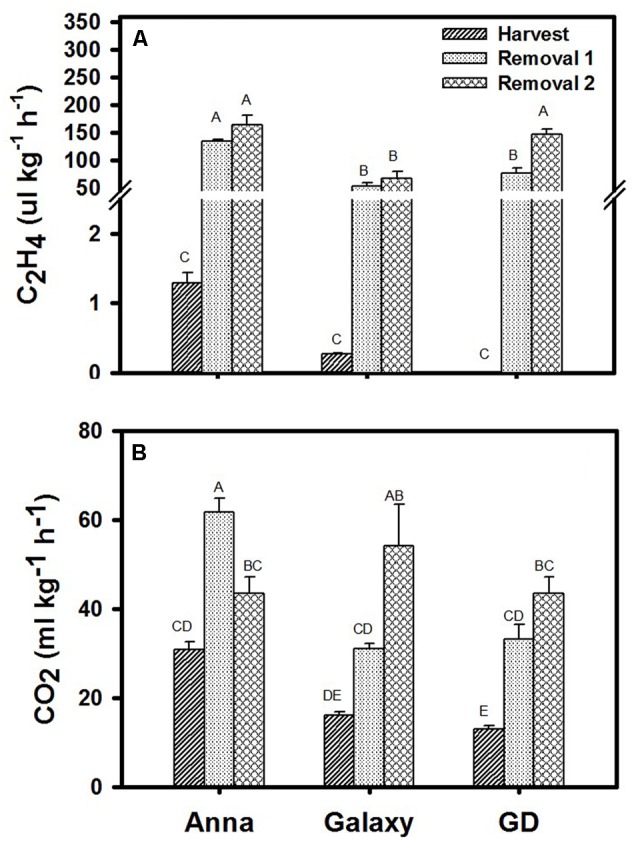
**(A)** Ethylene (C_2_H_4_) and **(B)** respiration (CO_2_ production) at harvest and storage (R1–R2). Each value represents the mean of 10 fruit and vertical bars indicate ±SE. Significance analysis was performed by comparing all cultivars and their stages collectively, using Tukey’s HSD pairwise comparison tests at *p* ≤ 0.05. Unlike letters represent significantly different groups.

Rates of ethylene (C_2_H_4_) and CO_2_ (respiration) production were determined at H, R1, and R2 for all three cultivars. Ethylene production rate increased following storage in all three cultivars where “Anna” produced significantly higher level of ethylene at harvest and following storage, compared to “Galaxy” and “GD” (**Figure 2A**). In parallel, “Anna” produced higher CO_2_ also at H, than the other two cultivars (**Figure 2B**). However, during storage, respiration rate of “Anna” exhibited a peak production at R1, while in the other two cultivars the respiration rate incremented gradually.

### Expression of Major *ACS* and *ACO* Genes at Harvest and Following Storage

Genes of *MdACS* and *MdACO* families are available in National Center for Biotechnology Information (NCBI) from several apple cultivars. However, it was not always clear to which chromosome location these sequences referred. We determined the chromosome location and the peptide length of all the available accessions (Supplementary Tables 1A,B). Total 19 genes were identified for *MdACS* (*1, 3A, 3B, 3C, 4, 5A, 5B*, and *6*–*17*), and 7 for *MdACO* (*1*–*7*) in the Genome Database for Rosaceae (GDR)^2^. A wide range expression analysis of all these genes was performed during fruit developmental, at harvest and following storage, in “Anna,” “Galaxy,” and “GD.” Six genes, from each family, *MdACS* (*MdACS1, 3a, 5B, 6, 8*, and *9*) and *MdACO* (*MdACO1*-5, 7) were expressed in either of the apple cultivars (Supplementary Tables 1A,B).

**FIGURE 3:**
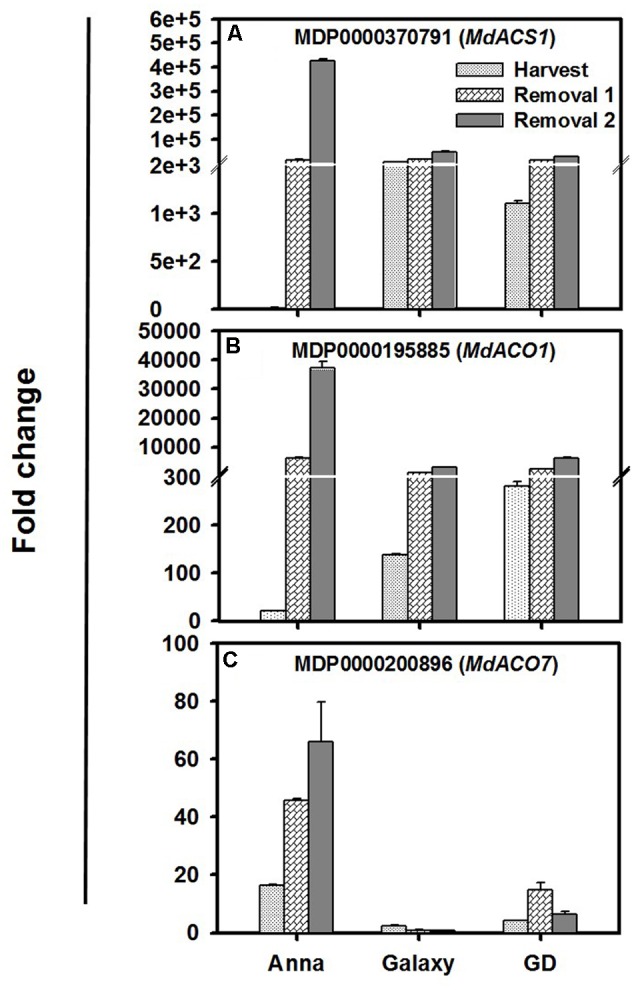
Expression profiles of **(A)** *MdACS1*, **(B)** *MdACO1*, and **(C)** *MdACO7* genes at harvest and storage (R1–R2). Expression of genes was calculated by 2^-ΔΔC_t_^ method, considering to the expression obtained at S1 stage and to house-keeping gene (HKG, actin), and is presented as relative fold change. Each value is the mean of three technical replicates ± SE. This is a representative of two independent replication.

The major ethylene biosynthesis gene, *MdACS1, MdACO1*, and *MdACO7* were expressed significantly higher during harvest and storage, in comparison to the other genes (**Figure 3**). At harvest the expression of *MdACS1* and *MdACO1* was lowest in “Anna”; however, their expression increased dramatically during storage (R1–R2) compared to “Galaxy” and “GD.” Examining the 5′ region of *MdACS1* revealed a 489 nucleotide section in “Anna,” which is a typical feature of *MdACS1-1* allele, related to higher gene expression and ethylene production (Supplementary Figure S3). *MdACO7* expressed the highest in “Anna,” and increased from H to R2, in contrast to both “Galaxy” and “GD” where expression was low.

### Dynamic Changes in Chlorophyll and Starch Levels during Fruit Development

**FIGURE 4:**
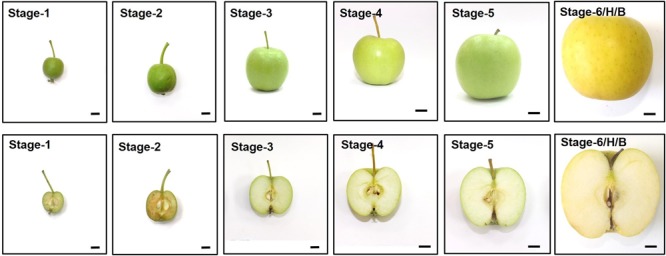
Developmental stages of “Golden Delicious (GD)” apple. Fruit at each stage were collected according to their size, and similar stages were also collected for “Anna” and “Galaxy.” Bar = 1 cm. See Supplementary Figure S1 for size and weight of the fruit from each stage of all three cultivars.

**FIGURE 5:**
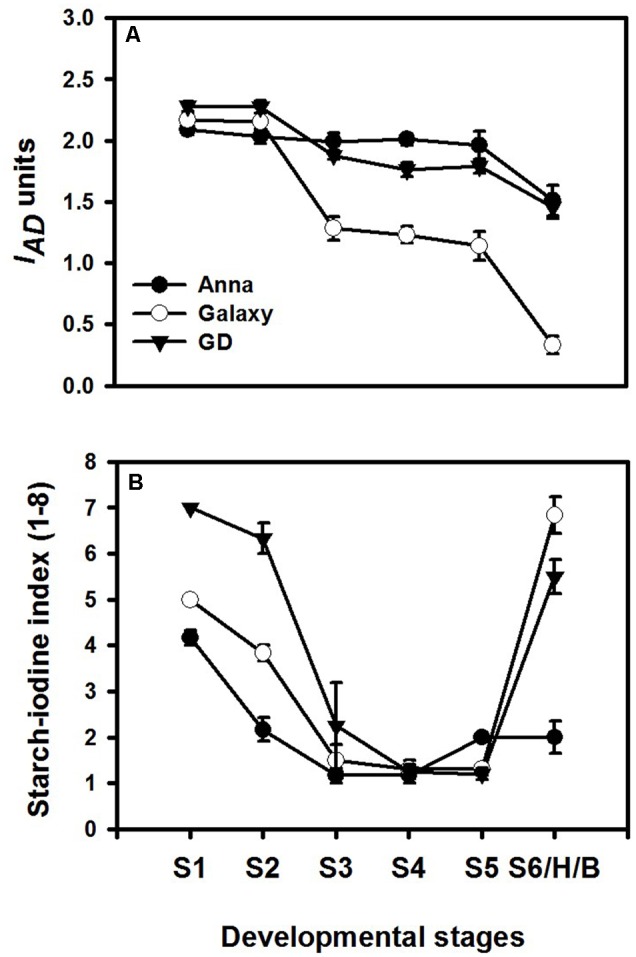
Chlorophyll and starch content at different stages of fruit development. **(A)** Chlorophyll content was measured by DA meter on two opposite sides of each fruit, and expressed as index of absorbance difference (*I*_AD_). Each value represents the mean of 10 fruit and vertical bars indicate ±SD. **(B)** Starch content was expressed as starch–iodine index, ranked on the scale of 1–8, where index-1 indicated highest starch content; however, index-8 corresponds to lowest starch content. Each value represents the mean of 10 fruit and vertical bars indicate ±SE.

Different developmental stages of all three cultivars; “Anna,” “Galaxy,” and “GD” were collected to follow changes in respiration, ethylene production rate, response to exogenous ethylene, expression of genes related to ethylene biosynthesis and response, in addition to developmental regulators. Fruit were harvested according to their size (S1–S6/H) (**Figure 4**) and the chlorophyll and starch content were monitored at these stages. Chlorophyll content was expressed as index of absorbance difference (*I*_AD_) (**Figure 5A**). The *I*_AD_ value of “Anna” remained similar throughout S1–S5, but declined significantly at S6. Both “Galaxy” and “GD” exhibited an earlier decline in *I*_AD_ at S3 and further at S6. At S6, a major decline was observed by 27, 84, and 36% for “Anna,” “Galaxy,” and “GD,” respectively, therefore, S6 was considered as the transition stage (Breaker, B), and indeed fruit were commercially harvested (H) at this stage. Starch content in the fruit was expressed as starch–iodine index (**Figure 5B**). In all cultivars, the starch content reaching the highest levels at S4 and in “Galaxy” and “GD” it remained high also at S5. In “Anna” slight decline was appeared at S5, which remained same at S6. On contrary, the levels of starch decline dramatically at S6 in both “Galaxy” and “GD.” Interestingly, “Anna” maintained higher starch levels during all developmental growth, compared to “Galaxy” and “GD.”

### Respiration and Ethylene Production Rates and the Response to Exogenous Ethylene during Fruit Development

**FIGURE 6:**
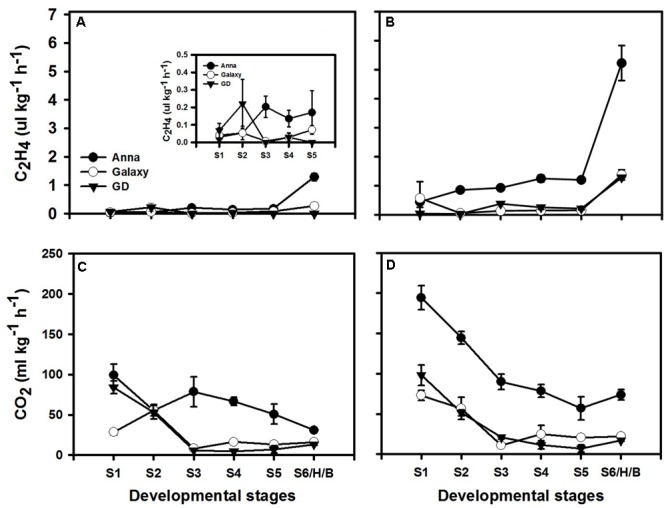
Ethylene (C_2_H_4_) and respiration (CO_2_ production) during different stages of fruit development. Graphs **(A)** and **(C)** represent normal course of ethylene and respiration, **(B)** and **(D)** represents response to exogenous ethylene (at 10 ppm). Each value represents the mean of 10 fruit and vertical bars indicate ±SE.

Since ethylene is known to enhance ethylene production in system II, but not in system I (Liu et al., 2015), we examined the respiration and ethylene production rates with (**Figures 6B,D**) or without (**Figures 6A,C**) exogenous ethylene treatment at S1–S6/H/B in all three cultivars. Non-treated “Galaxy” and “GD” fruit exhibited negligible ethylene production rate during development, while “Anna” had low ethylene production rate at S3 which was maintained in subsequent stages (**Figure 6A**). Both “Galaxy” and “GD” showed similar respiration rate featuring high rate at S1 with a decline at S3 and remaining negligible thereafter. In “Anna,” on the other hand, respiration rate remained high throughout development (**Figure 6C**).

Exogenous ethylene at all developmental stages induced the CO_2_ and ethylene production rates only in “Anna.” The ethylene production rate exhibited 6–14-fold increment for all stages and respiration rate exhibited an increase of 95–160% at S1–S2 and 12–17% at S3–S5. On the other hand, ethylene production increased in “Galaxy” only at S1 and S6, and in “GD” just at S6 (**Figures 6B,D**).

### Expression Analysis of Genes Related to Ethylene Biosynthesis, Response, and Ripening Regulators during Fruit Development

Since pre-climacteric ethylene and fruit developmental factors/mechanisms might affect fruit ripening (Alexander and Grierson, 2002; Giovannoni, 2004), the homolog of genes belonging to *MdACS* and *MdACO* families, encoding ETRs, regulatory components within the ethylene response pathway and few of the developmental regulators acting upstream of ethylene were identified in apple (**Table 1**). The expression pattern of these genes was determined during fruit development (S1–S6/H/B).

**Table 1:** Ethylene biosynthesis, signaling and other developmental regulator genes involved in apple fruit ripening.

S. No.	Apple genes	Gene ID (GDR)	Similar to tomato gene	References (function in tomato)
**Ethylene biosynthesis**				
(1)	*MdACS5B*	MDP0000435100	*SlACS1*	Barry et al., 2000
(2)	*MdACS6*	MDP0000133334	*SlACS3*	Yoshida et al., 2005
(3)	*MdACS8*	MDP0000250254	*SlACS3*	Yoshida et al., 2005
(4)	*MdACS9*	MDP0000166535	*SlACS3*	Yoshida et al., 2005
(5)	*MdACO2*	MDP0000200737	*SlACO4*	Nakatsuka et al., 1998
(6)	*MdACO3*	MDP0000725984	*SlACO1*	Barry et al., 1996
(7)	*MdACO4*	MDP0000251295	*SlACO1*	Barry et al., 1996
(8)	*MdACO5*	MDP0000453114	*SlACO1*	Barry et al., 1996
**Ethylene signaling**				
(9)	*MdCTR1*	MDP0000230308	*SlCTR1*	Leclercq et al., 2002
(10)	*MdETR1*	MDP0000557234	*SlETR1*	Lashbrook et al., 1998
(11)	*MdETR2*	MDP0000219737	*SlETR2*	Lashbrook et al., 1998
(12)	*MdERF1*	MDP0000128979	*SlERF1*	Li et al., 2007
(13)	*MdERF2*	MDP0000226115	*SlERF2*	Pirrello et al., 2006
(14)	*MdERF4*	MDP0000683814	*SlERF4*	Kim et al., 2013
(15)	*MdERF5*	MDP0000756341	*SlERF5*	Pan et al., 2012
(16)	*MdEIN2*	MDP0000152033	*SlEIN2*	Hu et al., 2010
(17)	*MdEIL1*	MDP0000423881	*SlEIL1*	Tieman et al., 2001
(18)	*MdEIL3*	MDP0000564884	*SlEIL3*	Tieman et al., 2001
(19)	*MdERS2*	MDP0000257135	*SlNr*	Hackett et al., 2000
**Developmental regulators**				
(20)	*MdSBP*	MDP0000271587	*SlSBP7*	Chen et al., 2010
(21)	*MdSBP2*	MDP0000249364	*SlSBP10*	Chen et al., 2010
(22)	*MdSBP7*	MDP0000181940	*SlSBP7*	Chen et al., 2010
(23)	*MdRIN*	MDP0000366022	*SlRIN*	Vrebalov et al., 2002
(24)	*MdCNR*	MDP0000180408	*SlCNR*	Thompson et al., 1999
(25)	*MdNOR*	MDP0000868419	*SlNOR*	Giovannoni, 2004
(26)	*MdHB1*	MDP0000737672	*SlHB1*	Lin et al., 2008
(27)	*MdPG1*	MDP0000326734	*SlPG1*	Sheehy et al., 1988
(28)	*MdAP2likeERF/TOE3*	MDP0000181606	*SlAP2*	Chung et al., 2010
(29)	*MdAP2*	MDP0000137561	*SlAP2*	Chung et al., 2010
(30)	*MdTAGL1*	MDP0000324259	*SlTAGL1*	Itkin et al., 2009
(31)	*MdFUL*	MDP0000289836	*SlFUL*	Bemer et al., 2012

**FIGURE 7:**
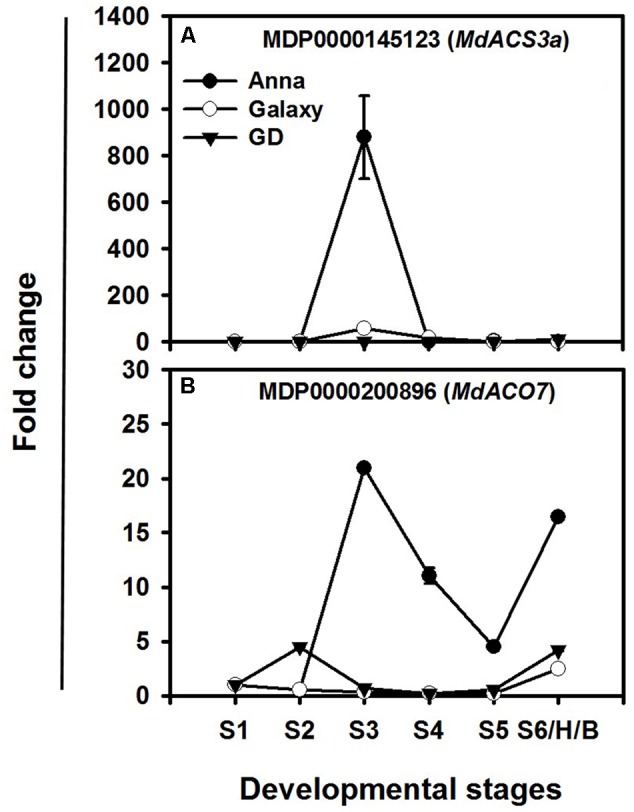
Expression profiles of **(A)** *MdACS3a* and **(B)** *MdACO7* genes during different stages of fruit development (S1–S6/H/B). Expression of genes at each stage was calculated by 2^-ΔΔC_t_^ method, considering to the expression obtained at S1 stage and to HKG (actin) and is presented as relative fold change. Each value is the mean of three technical replicates ± SE. This is a representative of two independent replication.

Among the ethylene biosynthesis genes, the highest increase in expression during fruit development occurs in *MdACS3a* and *MdACO7*, mainly in “Anna” (**Figures 7A,B**). “Anna” showed a rise at S3, with lower expression in other stages for *MdACS3a*, whereas “Galaxy” exhibited minor changes only at S3. *MdACO7* expressed throughout fruit development (S1–S6/H/B) of “Anna” with a peak at S3; however, both “Galaxy” and GD showed a lower change in expression of *MdACO7*.

**FIGURE 8:**
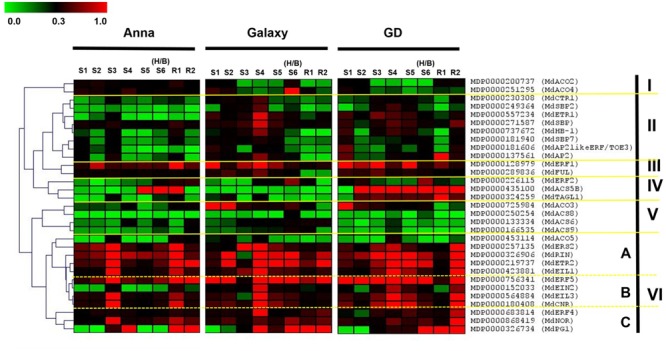
Heat map of the expression profile of genes involved in ethylene biosynthesis, ethylene response pathway, and developmental regulators, at different stage of fruit development (S1–S6/H/B) and at storage (R1–R2). The hierarchical clustering of genes is based on the Spearman correlation, which allows genes clustering according to their expression pattern and levels. Relative expression of the targeted genes are expressed by 2^-ΔC_t_^ method, considering expression in relation to HKG (actin). The green and red color corresponds to low and high expression, respectively.

The comprehensive gene expression profiling during fruit development (S1–S6/H/B) and also during storage (R1–R2) is presented in **Figure 8** (and also in Supplementary Figure S4). The hierarchical clustering of genes was based on the Spearman correlation and was clustered into six distinct clades (I–VI) according to their expression pattern and levels (**Figure 8**). Clade IV containing genes, *MdACS5B, MdTAGL1*, and *MdERF2* were highly expressed in “GD.” The expression of the genes of Clades III, V, VI A, and VI C was similar for all cultivars, except *MdFUL* and *MdACO3* which were higher in “GD” and “Galaxy” than in “Anna.” On the other hand, the expression of genes of Clade II (*MdCTR1, MdSBP, MdSBP2, MdSBP7, MdETR1, MdHB-1, MdAP2likeERF/TOE3, MdAP2*) and VI B (*MdERF5, MdEIN2, MdEIL3, MdCNR*) was higher in the two cultivars “Galaxy” and “GD” than in “Anna.” In contrary, clusters I included the genes *MdACO2* and *4* which were expressed higher in “Anna” than “Galaxy” and “GD.”

## Discussion

### “Anna” Cultivar Exhibiting Higher Lipid Oxidation-Related Autoluminescence and Ethylene Production Rate during Storage

The comparative storage capacity analysis of “Anna,” “Galaxy,” and “GD” apple fruit at their commercial harvest (S6) was most likely performed at a similar developmental stage. This stage might be similar to the “breaker” in tomato and it is supported by the fact that in all cultivars a major decline in chlorophyll content as expressed in *I*_AD_ was observed at stage 6 (**Figure 5**). The *I*_AD_ measurement has been established previously to identify the appropriate harvest time for different apple cultivars (Costamagna et al., 2013). In addition, auto-stimulatory response to exogenous ethylene of all cultivars at this stage further supports the notion that all cultivars were in a similar developmental stage (**Figure 6**). At this stage only “Galaxy” and “GD” expressed the *Polygalacturonase* (*PG1*) transcript (**Figure 8**), while it increased at storage in “Anna.” It seems that developmentally “Anna” might be slightly less mature than the others. Indeed, at this stage “Anna” contained higher starch than the other cultivars, which also might suggest lower maturity, but at least for starch, it has been suggested that in few cases its higher levels are not indicative of lower maturation (Watkins et al., 1993). Despite “Anna” having a lower *PG1* expression at harvest (**Figure 8**), its storage capacity was inferior to the other cultivars.

Juiciness and firmness determined at harvest and following storage confirmed the poor fruit quality of “Anna.” Although “Anna” at harvest was the juiciest among the cultivars, during storage, it lost its juiciness more than the other cultivars. It was suggested that lack of juiciness in apple is related to cell separation, preventing the release of cell content (Tu et al., 2000) and absorption of juice into pectin gel. More recently, it was suggested that it is related to cell wall degrading enzyme *pectin methyl esterases* (*PMEs*) which exhibited lower expression in mealy apple fruit throughout development in comparison to non-mealy fruit (Segonne et al., 2014), possibly leading to decreased cell-to-cell adhesion. A drastic declined in firmness occurred in “Anna,” further emphasizing the poor storage capacity of this cultivar. Our results are in accordance with the finding suggesting that early season apple cultivars are more prone to softening, compared to late season cultivars (Wiersma et al., 2007). Since “Anna” is grown at a lower altitude than the other cultivars, an environmental effect on this storage capacity cannot be excluded.

Low storage capacity of “Anna” was also reflected in high autoluminescence photon emission, which was highest in “Anna,” compared to “Galaxy” and “GD” following storage (**Figure 1F**). Since autoluminescence appears due to lipid oxidation under stress conditions (Birtic et al., 2011), we suggest that “Anna” was under oxidative stress. It is possible that the low storage capacity and the increase in autoluminescence of “Anna” resulted from higher respiration and ethylene production rates. High respiration is responsible for fast metabolism and a decline in fruit acidity, resulting from consumption of organic acid (Etienne et al., 2013). Indeed, TA was reduced significantly in “Anna” during storage where respiration rate was highest (**Figure 2**).

High ethylene production in “Anna” following storage coincided with higher expression of *MdACO1, 2, 4, 7*, and *MdACS1* (**Figure 3**). These genes, most likely, are involved in system II ethylene biosynthesis, as suggested for *MdACS1* (Dandekar et al., 2004) and *MdACO1* (Schaffer et al., 2007). We identified that “Anna” was homozygous *MdACS1-1/1-1* (Supplementary Figure S3), which was well correlated with the existence of homozygous *MdACS1-1/1-1* in higher ethylene producing apples (Sunako et al., 1999) and in early season apple cultivar (Oraguzie et al., 2004).

### “Anna” Cultivar Exhibits Properties of System II Throughout Fruit Development

Examining respiration and ethylene production rates throughout fruit development (S1–S5; **Figure 6**) revealed that “Anna” exhibited higher levels in comparison to “Galaxy” and “GD.” Furthermore, in response to exogenous ethylene treatment, “Anna” showed an ethylene-dependent positive feedback regulation with the induction of both ethylene and CO_2_ production throughout fruit development (**Figure 6**). Conversely, both “Galaxy” and “GD” remained unaffected by external ethylene at all stages prior to S6, indicated that system I operates prior to transition stage in these cultivars. Therefore, these results suggested the existence of a system II-like ethylene biosynthesis in “Anna,” where ethylene production is under auto-stimulatory control (Barry et al., 2000; Liu et al., 2015). A similar, system II-like ethylene biosynthesis has been reported in non-climacteric citrus fruit at young stage (Katz et al., 2004); however, unlike in “Anna” it was restricted to an early period of fruit development.

The higher ethylene and respiration in “Anna” was accompanied by higher expression of major ethylene biosynthesis genes, *MdACSO2, 4*, and *7* and particularly *MdACS3a* during early fruit development. It has been reported that the cultivars’ specific expression of *MdACS3a*, and existence of specific allele of this gene leads to high ethylene production in different apple cultivars (Wiersma et al., 2007; Wang et al., 2009; Varanasi et al., 2011; Bai et al., 2012).

In this study, we also examined genes within the ethylene response pathway and upstream transcription factors which control the ripening response (**Figure 8** and Supplementary Figure S4). Ethylene receptors and CTR kinase are negative regulators of the ethylene response (Liu et al., 2015). *MdETR1* showed a lower expression in all cultivars throughout development in comparison to *MdETR2* which fits with the observation that *MdETR2*, but not *MdETR1* is induced by ethylene. Nevertheless, *MdETR1, 2*, and *MdCTR1* exhibited lower expression in “Anna” in comparison to the other cultivars, during fruit development. The contribution of these negative regulators at the pre-breaker stage to “Galaxy” and “GD” fruit quality is still not clear.

### Developmental Regulation of Ripening in “Anna” Fruit

Homologs of negative (*AP2, MADS1*) or positive (*NOR, RIN, TAGL1, FUL1/2, CNR*) regulators of tomato ripening (Giovannoni, 2004; Karlova et al., 2014) were identified in apple, and most of them were expressed similarly in all three apple cultivars except, *MdFUL, MdAP2*, and *MdCNR*. These genes exhibited lower expression throughout development of “Anna,” in comparison to the other cultivars (**Figure 8** and Supplementary Figure S4). Fruitful (FUL) might have different function in apple and tomato, since in tomato reduced expression of *FUL1/2* inhibited mainly lycopene production, but not ethylene production, and in apple *MdMADS2* (*MdFUL*) was suggested to be involved in maintaining the apple fruit firmness (Cevik et al., 2010). Since expression of *MdFUL* was lower in “Anna” than in the other cultivars, we suggest that lower expression might be related to lower fruit firmness/higher mealiness (**Figure 1** and Supplementary Figure S2), but this should be further investigated. Lower expression of the *AP2* homolog enhanced ripening in tomato (Chung et al., 2010), and it is possible that it acts similarly in apple. The expression of *AP2* was low during early development (stages 2 and 3; 50–75 days after full bloom) of the cultivars “Galaxy” and “GD,” but increased later, however that of “Anna” remained low throughout development. Similar expression to that of “Galaxy” and “GD” was observed in the “Mondial Gala” apple cultivar (Costa et al., 2010). The expression pattern of *FUL* fits with its function as negative regulator. Colorless non-ripening is a SQUAMOSA SBP, critical for tomato fruit ripening, and the mutant *cnr*, exhibiting lower expression of the gene (Manning et al., 2006), has reduced ethylene production and mealy fruit, due to reduction in cell-to-cell adhesion (Thompson et al., 1999). Accordingly, we suggest that low expression of *MdCNR* in “Anna” might be responsible for the reduced expressible juice content and development of mealiness after harvest (**Figure 1D**). However, lower expression of *CNR* in “Anna” is also associated with higher ethylene production and faster firmness loss (**Figures 1, 2**). Since the nature of the mutation in “Anna” is still not clear it might be possible that additional processes are affected in “Anna.”

“Anna” also exhibited low expression of other *SBP, MdSBP, MdSBP2*, and *MdSBP7*. Few SBP transcription factors are known to bind the promotor of many genes responsible for maintaining copper homeostasis within the cell (Yamasaki et al., 2009) or assembly of mitochondrial electron transport chain complex IV subunits (Garcia et al., 2014). Moreover, SBP are also responsible for maintaining the levels of two isoforms of superoxide dismutase (SOD), iron SOD FeSOD, and copper SOD (CuSOD) under oxidative stress (Nagae et al., 2008). Since the expression of *MdSBP, MdSBP2*, and *MdSBP7* was lower in “Anna” than in “GD” and “Galaxy,” we postulate that these genes might be responsible for improper mitochondrial functioning or electron flow in this cultivar, which ultimately leads to high respiration rate (**Figure 6**). This might explain “Anna” higher autoluminescence, indicative of oxidative stress (**Figure 1F**).

Recently, the chromosome location of early bud break of “Anna” has been discovered including several SNPs in several genes (Trainin et al., 2016), however, so far, it is not clear if the low storage capacity of “Anna” is also localized to the same site. Taken together, this study provides new understanding on pre-climacteric events in “Anna” that might affect its ripening behavior and storage capacity.

## Conclusion

The poor storage capacity of “Anna” might be related to high lipid oxidation. This is associated not only with higher ethylene and respiration rates at harvest, but also with pre-climacteric system II-like characteristics. Modification in ethylene response genes and transcriptional regulators at pre-climacteric stage may be involved in this behavior in “Anna.”

## Author Contributions

VS carried out all the experiments, analyzed the datas, and write the manuscript. AW contributed in conducting the experiments. HF supervised the study and experiment, and contributed in the evaluation of manuscript. All authors read and approved the final manuscript.

## Conflict of Interest Statement

The authors declare that the research was conducted in the absence of any commercial or financial relationships that could be construed as a potential conflict of interest.

## Footnotes

1 http://www.fluidigm.com/user-documents

2 https://www.rosaceae.org
