# Corrigendum: Different Preclimacteric Events in Apple Cultivars with Modified Ripening Physiology

**DOI:** 10.3389/fpls.2017.01784

**Published:** 2017-10-16

**Authors:** Vikram Singh, Asya Weksler, Haya Friedman

**Affiliations:** Department of Postharvest Science of Fresh Produce, Agricultural Research Organization, Volcani Center, Bet Dagan, Israel

**Keywords:** “anna”, developmental regulators, ethylene biosynthesis, fruit development, MdFUL, system I/II, MdCNR, respiration

In the original article, Figure 8 and Supplementary Table 2 showed the number for MdRIN to be MDP0000326906. This is a mistake. The correct number is MDP0000366022.

The authors apologize for this error and state that this does not change the scientific conclusions of the article in any way.

## Conflict of interest statement

The authors declare that the research was conducted in the absence of any commercial or financial relationships that could be construed as a potential conflict of interest.

