# Supplementary material for: Different Preclimacteric Events in Apple Cultivars with Modified Ripening Physiology
Source: Front Plant Sci. 2017 Sep 5;8:1502. doi: 10.3389/fpls.2017.01502 (PMC5591845; doi:10.3389/fpls.2017.01502)
Supplement: Supplementary file 1 [file Table_1.DOCX]

Supplementary Table 1. *Malus domestica* (A) *ACS* and (B) *ACO* genes and their chromosome location. Genes were browsed in apple genome database, Genome Database for Rosaceae (GDR, [https://www.rosaceae.org](https://www.rosaceae.org/)). Each gene is characterize according to their chromosomal location, peptide length and accession numbers. The expression behavior of genes investigated in our study is specified by + or – symbols, denoting expressed and non-expressed genes, respectively.

Supplementary Table 1 A

| **S. No.** | **Gene name** | **Expression**  **(in fruit)** | **Gene ID**  **(GDR)** | **Chromosome location** | **Accessions**  **(NCBI)** | **Cultivar** | **Peptide**  **length** |
| --- | --- | --- | --- | --- | --- | --- | --- |
| **1.** | *MdACS1* | + | MDP0000370791 | Unanchored: 684,857..686,705 |  | Golden Delicious | 473 |
|  |  |  |  | - | AB010102.1 | Golden Delicious | 473 |
|  |  |  |  | - | AF312737.1 | Granny Smith | 460 |
|  |  |  |  | Unanchored: 685,646..687,281 | AJ011518.1 | Golden Delicious | 403 |
|  |  |  |  | Unanchored: 685,083..687,484 | L31347.1 | Golden Delicious | 473 |
|  |  |  |  | - | U03294.1 | Golden Delicious | 464 |
|  |  |  |  | Unanchored: 685,083..687,484 | U89156.1 | Golden Delicious | 473 |
|  | *MdACS1-1* |  |  | - | AY062129.1 | McIntosh | 22 |
| **2.** | *MdACS3A* | + | MDP0000145123 | chr13: 32,030632..32,032,337 |  | Golden Delicious | 446 |
|  | *MdACS3a* |  |  | chr13: 32,030,560..32,032,337 | AB243060.2 | Golden Delicious | 446 |
|  | *MdACS3a* |  |  | - | AY821542.1 | Granny Smith | 408 |
|  | *MdACS3a* |  |  | chr13: 32,030,560..32,032,522 | U73816.1 | MacIntosh | 446 |
|  | *MdACS3a-1* |  |  | chr13: 32,030632..32,032,337 | JF833308.1 | Golden Delicious | 446 |
|  | *MdACS3a-2* |  |  | chr13: 32,030632..32,032,337 | JF833309.1 | Narihoko | 446 |
| **3.** | *MdACS3B* | - | MDP0000406217 | chr2: 5756294..5757868 |  | Golden Delicious | 393 |
|  |  |  |  | - | AB243061 | Golden Delicious | 445 |
| **4.** | *MdACS3C* | - | MDP0000874578 | chr2: 5735557..5737215 |  | Golden Delicious | 447 |
|  |  |  |  | chr2: 5735454..5737200 | AB243062 | Golden Delicious | 446 |
| **5.** | *MdACS4* | - | MDP0000262827 | chr1: 9880090..9882296 |  | Golden Delicious | 495 |
| **6.** | *MdACS5A* | - | MDP0000923426 | chr2: 33731213..33733562 |  | Golden Delicious | 491 |
|  |  |  |  | chr2: 33730797..33733542 | AB034992.1 | - | 487 |
| **7.** | *MdACS5B* | + | MDP0000435100 | chr7: 14188083..14190401 |  | Golden Delicious | 487 |
|  |  |  |  | chr7: 14188043..14190716 | AB034993.1 | - | 487 |
| **8.** | *MdACS6* | + | MDP0000133334 | chr1:24222638..24225153 |  | Golden Delicious | 559 |
| **9.** | *MdACS7* | - | MDP0000508068 | chr1:24297643..24300839 |  | Golden Delicious | 345 |
| **10.** | *MdACS8* | + | MDP0000250254 | chr6:10620365..10622922 | - | Golden Delicious | 500 |
| **11.** | *MdACS9* | + | MDP0000166535 | chr3:17711719..17716945 | - | Golden Delicious | 662 |
| **12.** | *MdACS10* | - | MDP0000413933 | chr9:9966399..9982296 | - | Golden Delicious | 663 |
| **13.** | *MdACS11* | - | MDP0000454938 | chr15:1187928..1190212 | - | Golden Delicious | 429 |
| **14.** | *MdACS12* | - | MDP0000321088 | chr2:19705227..19707071 | - | Golden Delicious | 462 |
| **15.** | *MdACS13* | - | MDP0000123248 | chr7:14200818..14204430 | - | Golden Delicious | 503 |
| **16.** | *MdACS14* | - | MDP0000232577 | chr15:1160786..1165188 | - | Golden Delicious | 567 |
| **17.** | *MdACS15* | - | MDP0000408853 | chr1:24323482..24328019 | - | Golden Delicious | 292 |
| **18.** | *MdACS16* | - | MDP0000308887 | chr15:1183194..1185615 | - | Golden Delicious | 460 |
| **19.** | *MdACS17* | - | MDP0000265383 | chr1:22664736..22665994 | - | Golden Delicious | 108 |

Supplementary Table 1 B

| **S. No.** | **Gene name** | **Expression (in fruit)** | **Gene ID (GDR)** | **Chromosome location** | **Accessions**  **(NCBI)** | **Cultivar** | **Peptide length** |
| --- | --- | --- | --- | --- | --- | --- | --- |
| **1.** | *MdACO1* | + | MDP0000195885 | chr10: 32,199,150..32,200,787 | - | Golden Delicious | 314 |
|  | *MdACO1-1* |  |  | chr10: 32,199,178..32,199,737 | AY598766.1 | Fuji | 92 |
|  | *MdACO1-2* |  |  | chr10: 32,199,195..32,199,997 | DQ439791.1 | Golden Delicious | 174 |
|  | *MdACO1-2* |  |  | chr10: 32,199,178..32,199,737 | AY598767.1 | Mondial Gala | 92 |
|  | *MdACO1-2* |  |  | chr10:32199150..32199380 | DQ439792.1 | Golden Delicious | 231 |
|  | *MdACO1a* |  |  | chr10:32199172..32200766 | JQ675679.1 | Jonathan | - |
|  | *MdACO1b* |  |  | - | JQ675680.1 | Jonathan | 301 |
|  | *MdACO1c* |  |  | - | JQ675681.1 | Dukat | 300 |
|  |  |  |  | chr10: 32,199,150..32,200,787 | DQ137850.1 | - | 314 |
|  |  |  |  |  | X61390.1 | Golden Delicious | - |
|  |  |  |  |  | X98627.1 | Fuji | - |
| **2.** | *MdACO2* | + | MDP0000200737 | chr5: 801,646..803,236 | - | Golden Delicious | 330 |
|  |  |  |  | chr5: 802,401..803,143 | DQ438990.1 | Golden Delicious | 174 |
|  |  |  |  | chr5: 801,588..803,375 | AF015787.1 | Fuji | 330 |
| **3.** | *MdACO3* | + | MDP0000725984 | chr9: 7,856,139..7,857,630 | - | Golden Delicious | 322 |
|  |  |  |  | chr9: 7,856,080..7,857,824 | AB086888.1 | Fuji | 322 |
| **4.** | *MdACO4* | + | MDP0000251295 | chr17: 8,476,210..8,477,586 | - | Golden Delicious | 322 |
| **5.** | *MdACO5* | + | MDP0000453114 | chr9: 7,892,904..7,894,395 | - | Golden Delicious | 322 |
|  |  |  |  | chr9: 7,856,080..7,857,824 | AB086888.1 | Fuji | 322 |
| **6.** | *MdACO6* | - | MDP0000025650 | chr1: 465,389..466,698 | - | Golden Delicious | 298 |
| **7.** | *MdACO7* | + | MDP0000200896 | chr15:14,547,480..14,549,801 | - | Golden Delicious | 348 |
