# Supplementary material for: Different Preclimacteric Events in Apple Cultivars with Modified Ripening Physiology
Source: Front Plant Sci. 2017 Sep 5;8:1502. doi: 10.3389/fpls.2017.01502 (PMC5591845; doi:10.3389/fpls.2017.01502)
Supplement: Supplementary file 2 [file Table_2.DOCX]

Supplementary Table. 2 Primers used for gene expression analysis-

| **S. No.** | **Gene name** | **Gene ID (GDR)** | **Accession number** | **Tomato/ Arabidopsis homolog** | **Forward primer (5'-3')** | **Reverse primer (5'-3')** |
| --- | --- | --- | --- | --- | --- | --- |
| **1** | *MdACO1* | MDP0000195885 | DQ137850.1 | - | TGAGCTTTTGGACACTGTGG | ACATCGTCGAGGCCTTTG |
| **2** | *MdACO2* | MDP0000200737 | AF015787.1 | - | TTCTGGCCTCAAATTCCAAG | CATCGGACGGCTCTCAGAC |
| **3** | *MdACO3* | MDP0000725984 | AB086888 | - | CACAGTGGAGAGGCTGACAA | CTTCTGTCTGAACACCCTCAAG |
| **4** | *MdACO4* | MDP0000251295 | - | - | TCTTGGACTGGAACAAGGGTA | TTGTCATTGTTGGGACATGG |
| **5** | *MdACO5* | MDP0000453114 | - | - | GCACCACAAGAACAAATTAACC | CCATTGAGGCTCTCCAAGTT |
| **6** | *MdACO7* | MDP0000200896 | - | - | GGTGGAAAGGGTCCTTCTGT | CACCAGCATCAGTGTGCTCT |
| **7** | *MdACS1* | MDP0000370791 | U89156 | - | GAGCTTGCTCTCTTCCAAGATT | CGGTGAGCACTAAGTGGTTG |
| **8** | *MdACS3a* | MDP0000145123 | AB243060 | - | TGTCTGGCTTTAGAGAAAATGC | CAAATTTGGCTCTTCCTCCTC |
| **9** | *MdACS5B* | MDP0000435100 | AB034993 | - | CAGAAGGCAAACATCAGAGTAAAGG | TTATGAAACTTGGCTGACTGAACAC |
| **10** | *MdACS6* | MDP0000133334 | - | - | AGTGGATTAATTCGCTCTTATGGTG | GGAACATCCTCTGGAGTCAAAGTAG |
| **11** | *MdACS8* | MDP0000250254 | - | - | GGCTCCGTGCAATGTACCTA | GGATTAACCCGCTCATGTCA |
| **12** | *MdACS9* | MDP0000166535 | - | - | CCAGCGCTATCTACTCCTTTAACAA | TACATTCCACGAAGCCTTTCTCTAC |
| **13** | *MdERF1* | MDP0000128979 | KC128856.1 | - | ATGACCTGGTGGCATATCAG | CACCGTAGCAAACAACACAC |
| **14** | *MdERF2* | MDP0000226115 | KC128857.1 | - | TATGCTGGCAATTGGCGAGC | ATGACCAATCCCGCACTCAC |
| **15** | *MdERF4* | MDP0000683814 | - | - | AGCAGCAGCACAATGACATC | AGGGGATGTTGAAGAAGCAC |
| **16** | *MdERF5* | MDP0000756341 | - | - | GCTGTTCAAACCGCTCTCC | TGGGTCGGGGTACTCATTTA |
| **17** | *MdEIN2* | MDP0000152033 | DQ845461.1 | - | TAGGCAGCCTTTTGAGCAAT | TGCAACTGAAGTTGTTTCTTGA |
| **18** | *MdEIL1* | MDP0000423881 | KC128858.1  GU732484.1 | - | GTTCGATGCTTCGGGACTT | ACCTGACTGGTTCACTGGTTG |
| **19** | *MdEIL3* | MDP0000564884 | KC128860.1  GU732486.1 | - | GAAGTGTGGAGAAGTTGACGTGG | CTTCATCACTATAATCCTCCTCTGC |
| **20** | *MdETR1* | MDP0000557234 | AY040228.1 | - | TTGGCCTGTGAAGAGCAGT | TGCAAACCATGTAGAGCCAT |
| **21** | *MdETR2* | MDP0000219737 | DQ847145 | - | GTTGTGACGCGGAAAATGC | AATCCAGATGAAACGGCAGTTAC |
| **22** | *MdERS2* | MDP0000257135 | AB213028 | - | GCTTGTTAAGGTTGGAAGAAATCTG | CGGCATCGTTGAGTGTTACATT |
| **23** | *MdCTR1* | MDP0000230308 | DQ847148 | - | ACAAGATTTTCATGCCGAAC | TATGGACAAGTTTGGAGGCT |
| **24** | *MdPG1* | MDP0000326734 | L27743 | - | CGCACAACAAATCCATGTCATAT | ACCGTGAGACAGGAAGCTTGA |
| **25** | *MdAP2like*  *ERF/TOE3* | MDP0000181606 | - | AT5G67180 | CCAACTCGTCCGTTGTCAAT | CCTGGACCTTGAGAATACCG |
| **26** | *MdHB-1* | MDP0000737672 | - | AT3G01470 | GGCCGTCTTTTCTTTGAGC | GATCTTGGCCCTCGAAAAAC |
| **27** | *MdNOR (NAC5)* | MDP0000868419 | HM122668.1 | Solyc10g006880 | CTCACAACAGCAACAGCACC | CGGAGGTGGCCTTTTTCTTG |
| **28** | *MdSBP* | MDP0000271587 | - | - | GCACCGTTTAGACCAACCAC | CCAAGAGATCTCCCCAATCC |
| **29** | *MdSBP2* | MDP0000249364 | - | - | CACCCATCAAGGCTGTCTTC | ACGAGCCATCCCATGTTAAA |
| **30** | *MdSBP7* | MDP0000181940 | - | - | CGATGACTTGTCGATCTCGT | CCGCTTCCTTATCCGACTC |
| **31** | *MdCNR (SBP)* | MDP0000180408 | - | Solyc02g077920 / AT2G33810 | AATTTTCGTGGTGTGAAGGTG | GGTGTAGAATTCAATGGACTAGCA |
| **32** | *MdAP2* | MDP0000137561 | GU983666.1  AF332215.2  GU732464.1 | Solyc03g044300.2.1 | TGTGGGATCTCAATGACTCG | GACCCGTTTCCTCTTTTCCT |
| **33** | *MdRIN* | MDP0000326906 | U78947.1-MADS1  AJ001681.1-MADS8 | Solyc05g012020.2 | TGAGCATGTGCTGATGAATCTG | GCAAAGCCCTCTGTAACTAACG |
| **34** | *MdTAGL1/SHP* | MDP0000324259 | HM122598.1- MADS14  HM122598.1- MADS14 | AT3G58780(SHP1)  AT2G42830(SHP2) | GCAAATCAAGCACCTGAGAG | TTGCAGAAGGTGACTTGTCG |
| **35** | *MdFUL* | MDP0000289836 | DQ205652.1-MADS2  U78948.1 | Solyc06g069430.2 (FUL1/TDR4) | AAGGGTGCAGCTGAAGAGAA | TCAAAGCAACCTCAGCATCA |
| **36** | *Mdactin* | MDP0000170174 | AB638619.1 | - | GGCTCTATTCCAACCATCCA | TAGAAGCAGTGCCACCACAC |
