# Supplementary figures and images for: Different Preclimacteric Events in Apple Cultivars with Modified Ripening Physiology
Source: Front Plant Sci. 2017 Sep 5;8:1502. doi: 10.3389/fpls.2017.01502 (PMC5591845; doi:10.3389/fpls.2017.01502)

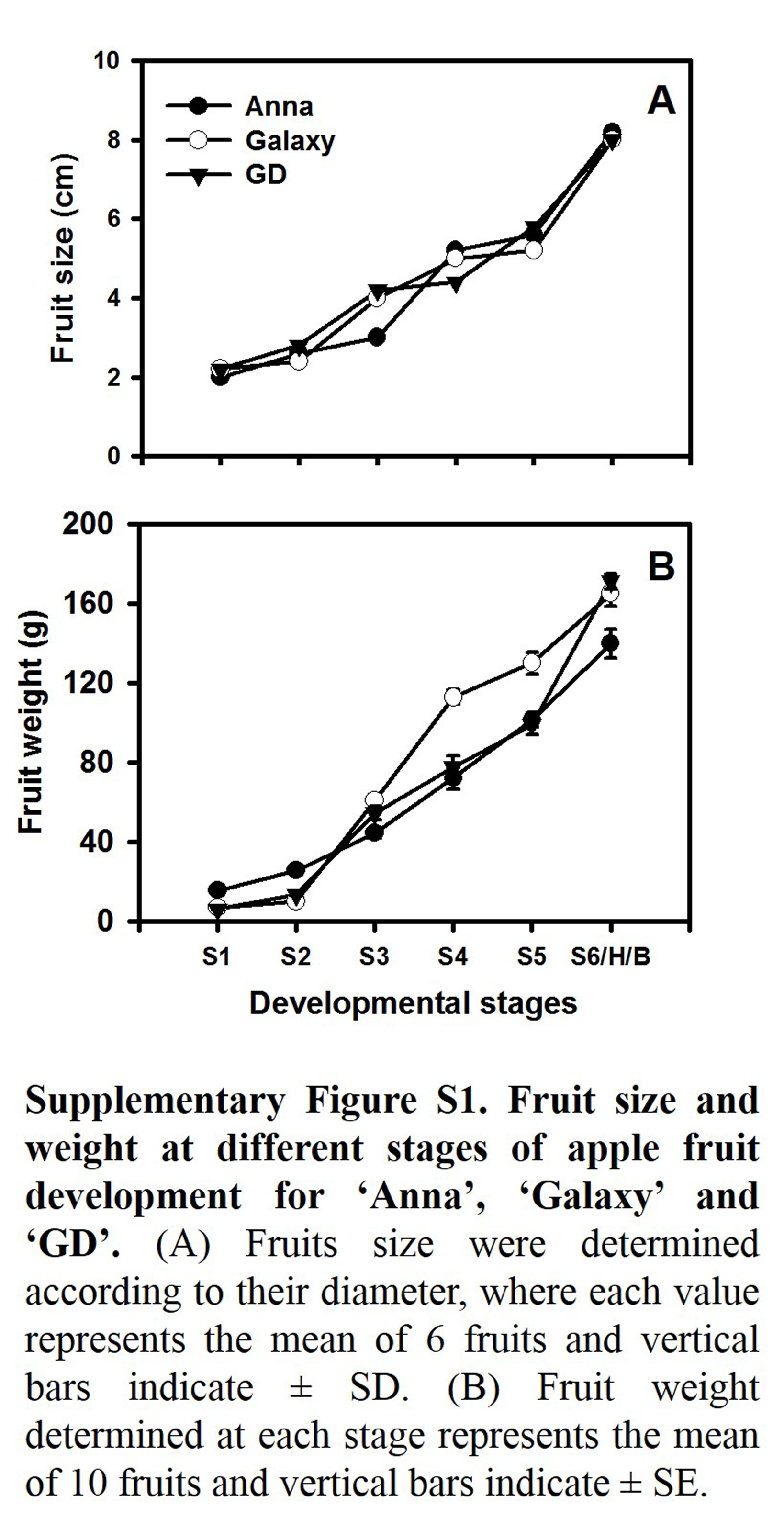

Supplement: Supplementary file 3 [file Image_1.TIF]

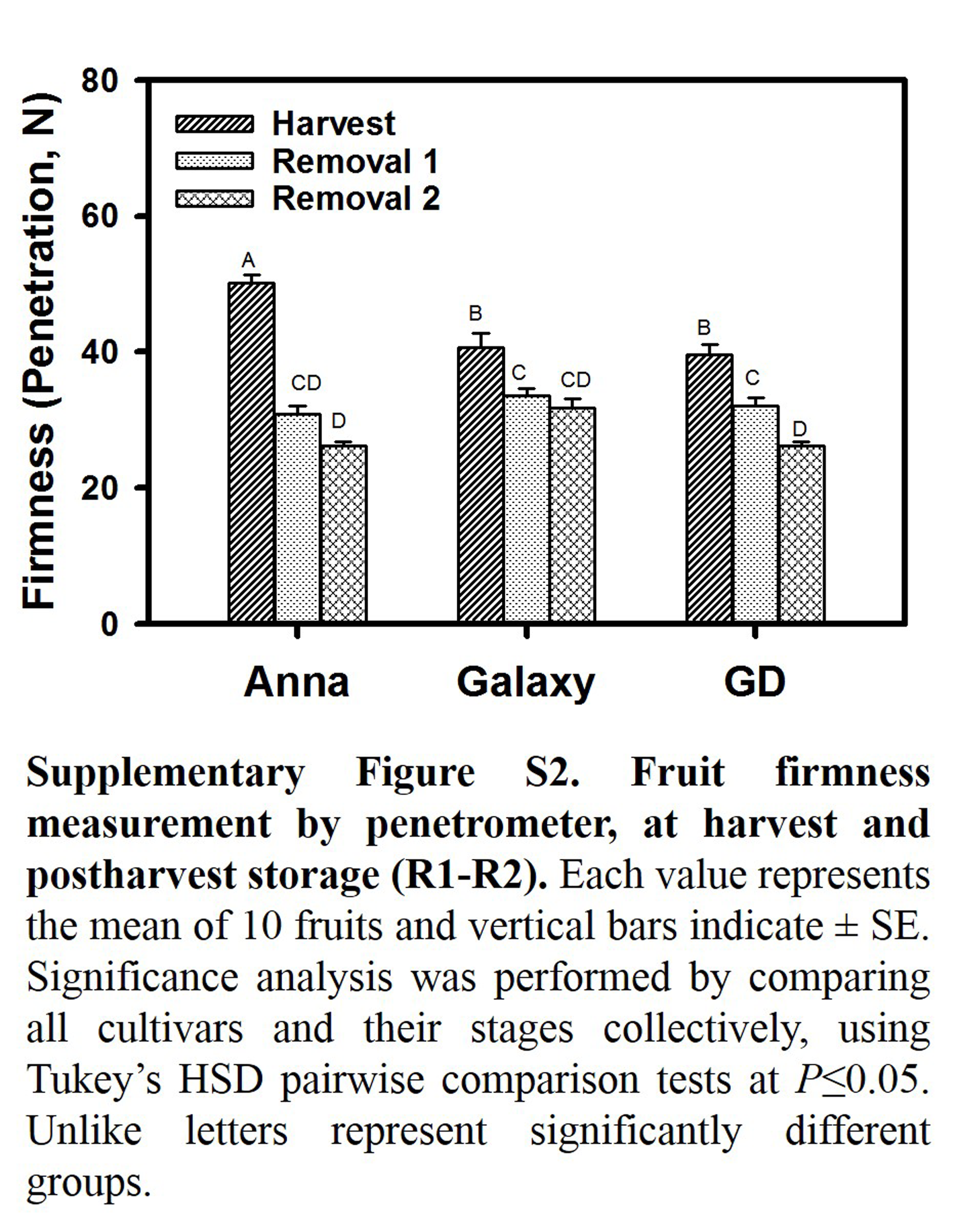

Supplement: Supplementary file 4 [file Image_2.TIF]

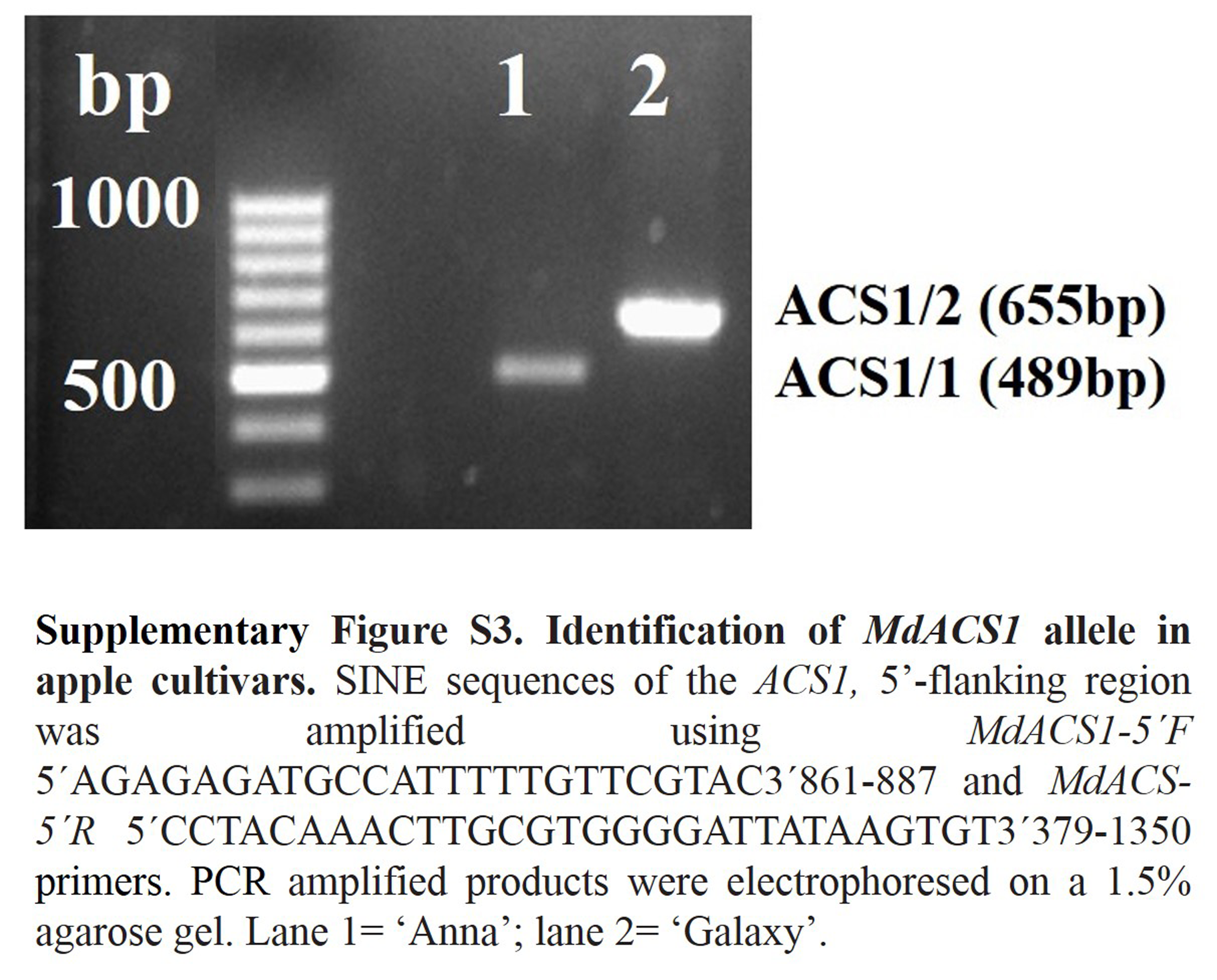

Supplement: Supplementary file 5 [file Image_3.TIF]
