# Supplementary material for: Different Preclimacteric Events in Apple Cultivars with Modified Ripening Physiology
Source: Front Plant Sci. 2017 Sep 5;8:1502. doi: 10.3389/fpls.2017.01502 (PMC5591845; doi:10.3389/fpls.2017.01502)
Supplement: Supplementary file 6 [file Image_4.PDF]

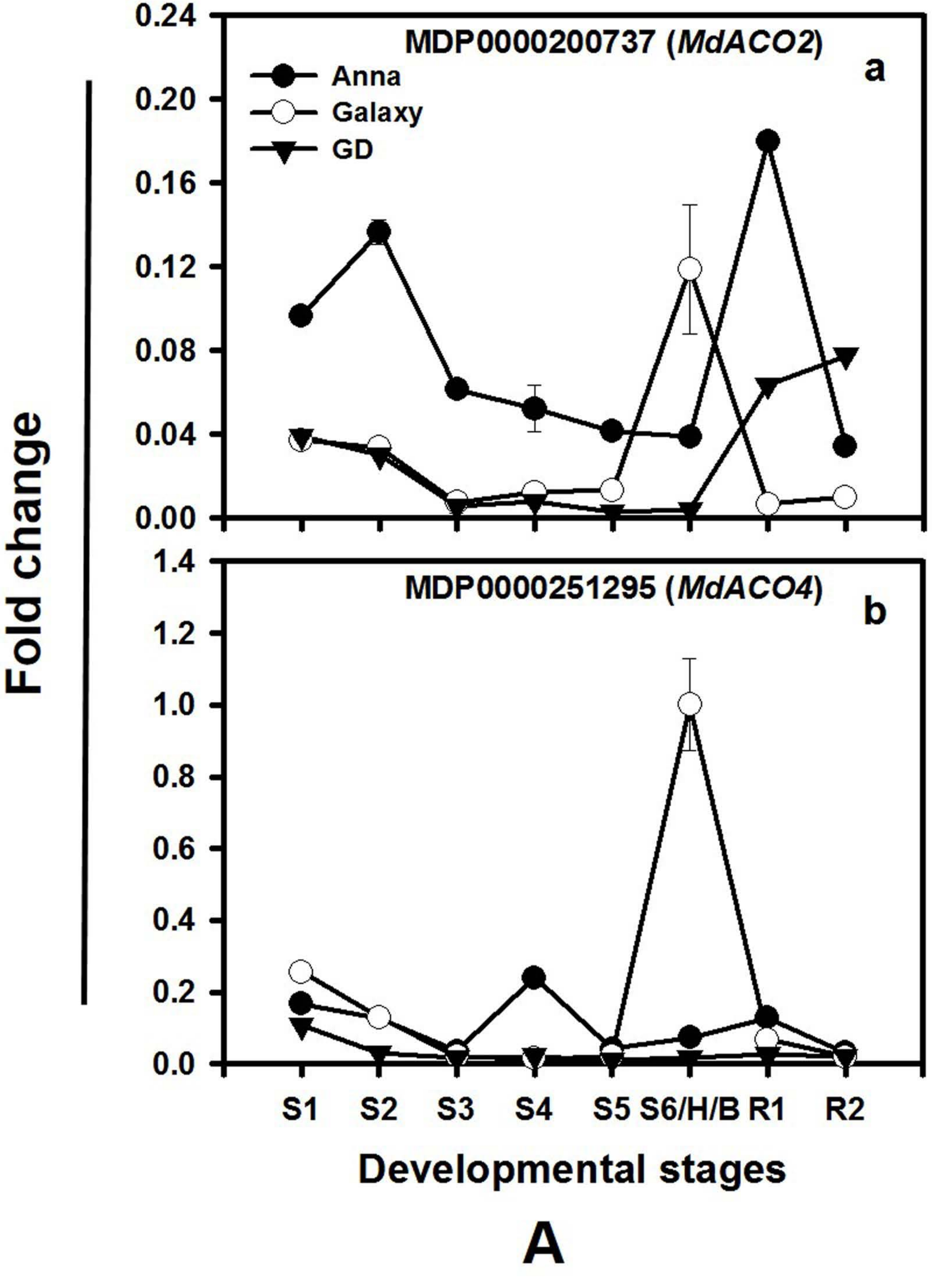

Fold change
MDP0000200737 (*MdACO2*)
a
Anna
Galaxy
GD
0.24
0.20
0.16
0.12
0.08
0.04
0.00
MDP0000251295 (*MdACO4*)
b
1.4
1.2
1.0
0.8
0.6
0.4
0.2
0.0
S1
S2
S3
S4
S5
S6/H/B
R1
R2
Developmental stages
A

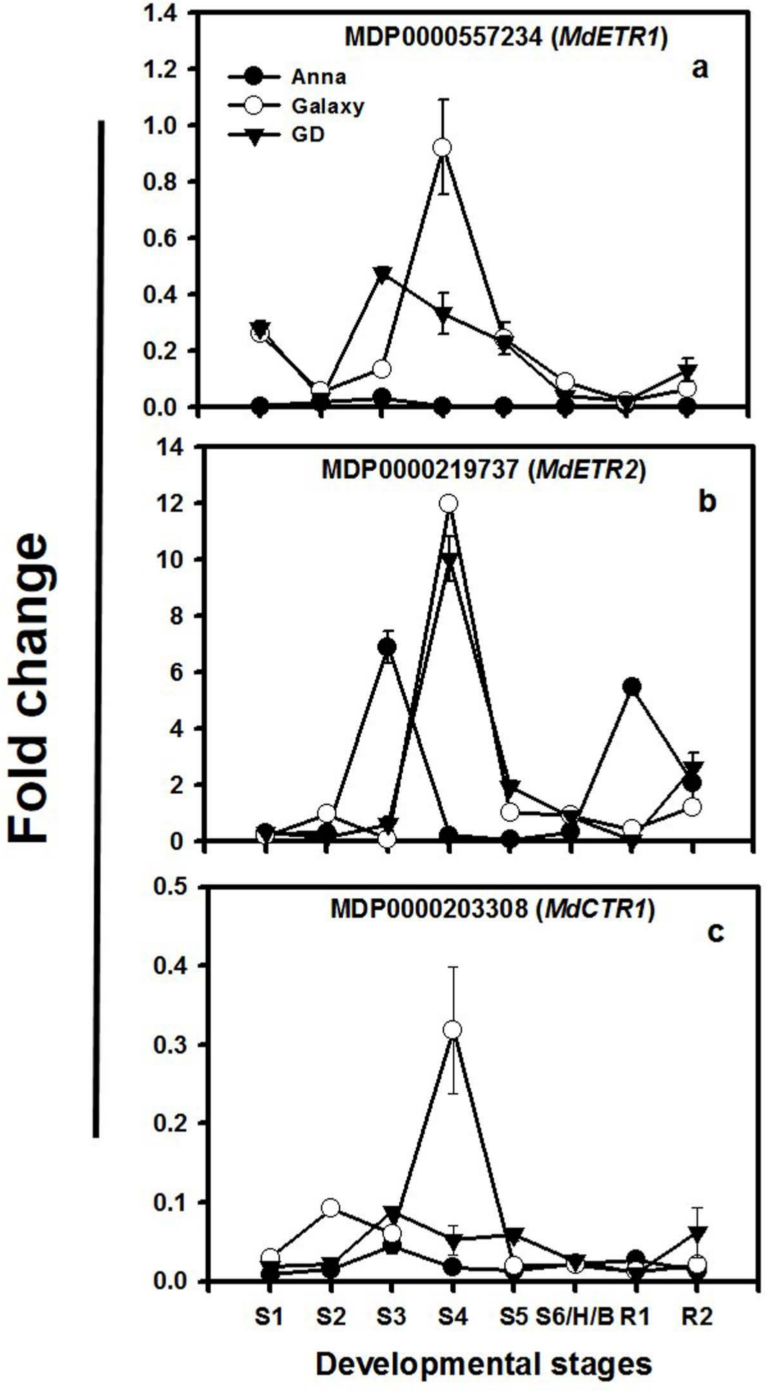

Fold change
MDP0000557234 (MdETR1)
a
Anna
Galaxy
GD
1.4
1.2
1.0
0.8
0.6
0.4
0.2
0.0
MDP0000219737 (MdETR2)
b
14
12
10
8
6
4
2
0
MDP0000203308 (MdCTR1)
c
0.5
0.4
0.3
0.2
0.1
0.0
S1
S2
S3
S4
S5
S6/H/B
R1
R2
Developmental stages

B

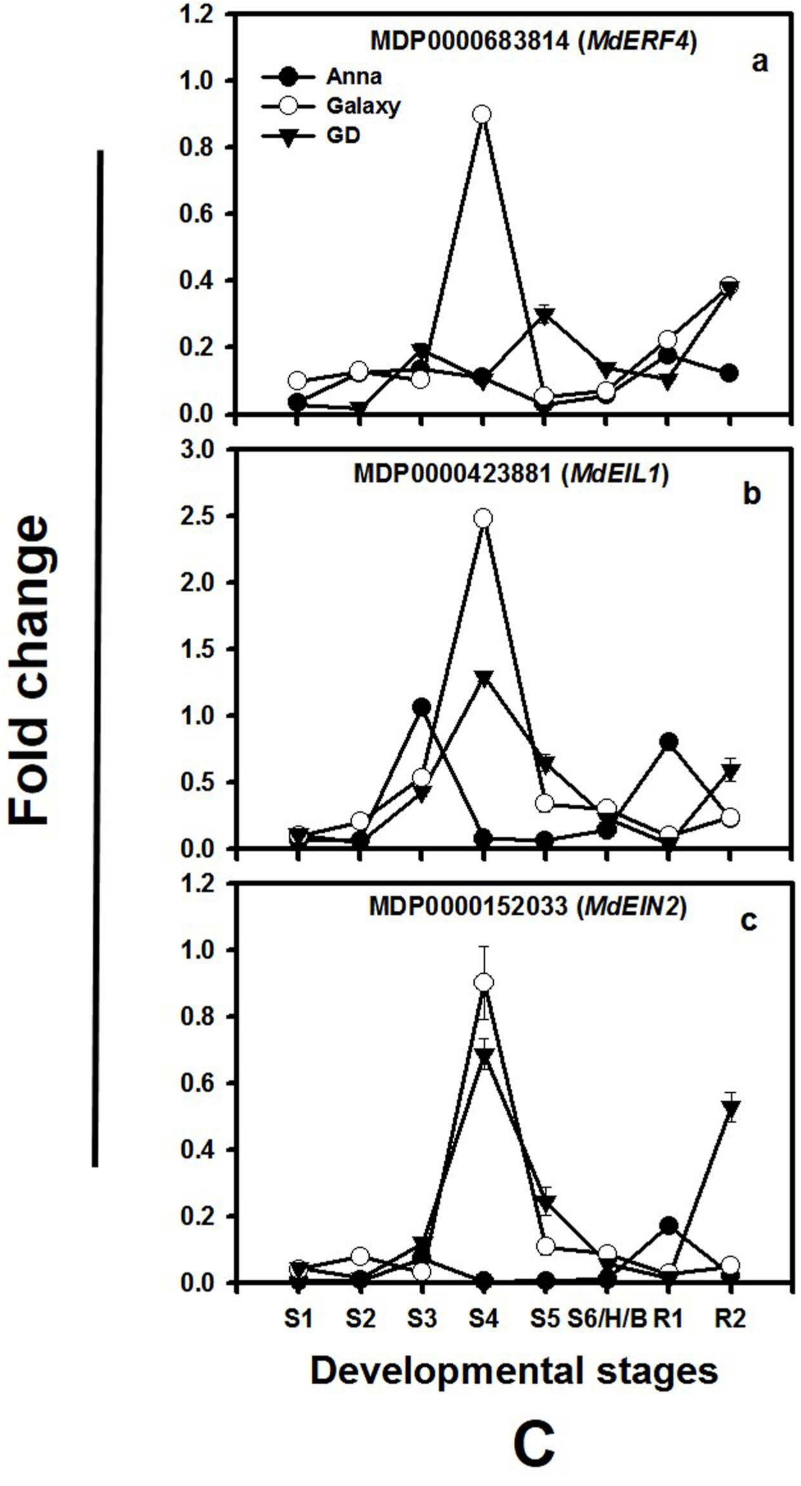

Fold change
MDP0000683814 (MdERF4)
a
Anna
Galaxy
GD
MDP0000423881 (MdEIL1)
b
MDP0000152033 (MdEIN2)
c
S1
S2
S3
S4
S5
S6/H/B
R1
R2
Developmental stages
C

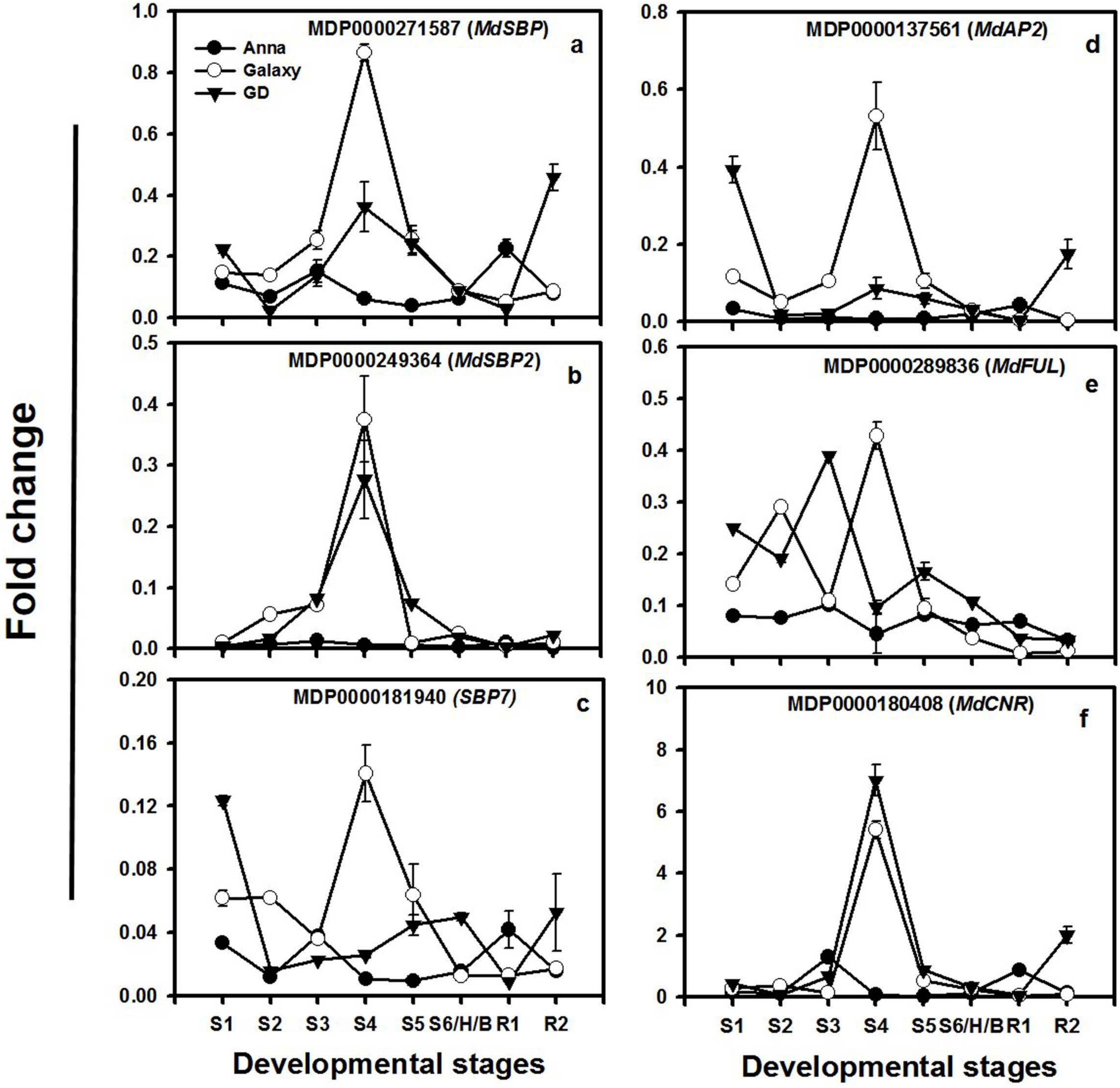

Fold change
MDP0000271587 (MdSBP)
a
Anna
Galaxy
GD
MDP0000249364 (MdSBP2)
b
MDP0000181940 (SBP7)
c
MDP0000137561 (MdAP2)
d
MDP0000289836 (MdFUL)
e
MDP0000180408 (MdCNR)
f
S1 S2 S3 S4 S5 S6/H/B R1 R2
Developmental stages

D

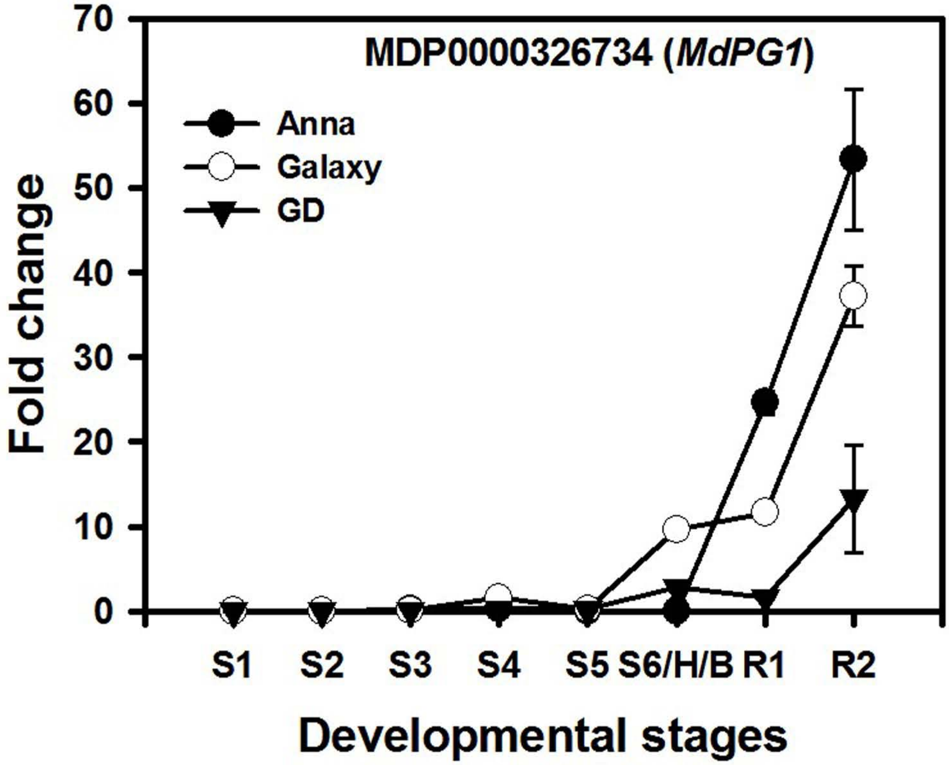

E

Supplementary Figure S4. Expression profiles of (A) (a) *MdACO2* (b) *MdACO4*, (B) (a) *MdETR1*, (b) *MdETR2* (c) *MdCTR1*, (C) (a) *MdERF4*, (b) *MdEIL1*, (c) *MdEIN2*, (D) (a) *MdSBP*, (b) *MdSBP2*, (c) *MdSBP7*, (d) *MdAP2*, (e) *MdFUL* (f) *MdCNR* (E) *MdPG1* genes during different stages of fruit development (S1-R2). Expression of genes at each stage was calculated by $2^{-\Delta Ct}$ method, considering expression in relation to house-keeping gene (HKG, actin) and is presented as relative fold change. Each value is the mean of three technical replicates ± SE. This is a representative of 2 independent replication.
